# Contamination and carryover free handling of complex fluids using lubricant-infused pipette tips

**DOI:** 10.1038/s41598-022-18756-x

**Published:** 2022-08-25

**Authors:** Amid Shakeri, Hanie Yousefi, Noor Abu Jarad, Samer Kullab, Dalya Al-Mfarej, Martin Rottman, Tohid F. Didar

**Affiliations:** 1 Department of Mechanical Engineering, McMaster University, 1280 Main Street West, Hamilton, ON L8S 4L7 Canada; 2 Leslie Dan Faculty of Pharmacy, University of Toronto, 144 College Street, Toronto, ON M5S 3M2 Canada; 3 School of Biomedical Engineering, McMaster University, 1280 Main Street West, Hamilton, ON L8S 3L8 Canada; 4 Department of Microbiology and Innovative Biomarkers Platform, GH Université Paris Saclay, Hôpital Raymond Poincaré (APHP), Garches, France; 5 Laboratory of Infection and Inflammation U1173, School of Medicine Simone Veil Versailles Saint-Quentin-en-Yvelines University, Montigny le Bx, France

**Keywords:** Biotechnology, Chemistry, Materials science

## Abstract

Cross-contamination of biological samples during handling and preparation, is a major issue in laboratory setups, leading to false-positives or false-negatives. Sample carryover residue in pipette tips contributes greatly to this issue. Most pipette tips on the market are manufactured with hydrophobic polymers that are able to repel high surface tension liquids, yet they lack in performance when low surface tension liquids and viscous fluids are involved. Moreover, hydrophobicity of pipette tips can result in hydrophobic adsorption of biomolecules, causing inaccuracies and loss in precision during pipetting. Here we propose the use of lubricant-infused surface (LIS) technology to achieve omniphobic properties in pipette tips. Using a versatile and simple design, the inner lumen of commercially available pipette tips was coated with a fluorosilane (FS) layer using chemical vapor deposition (CVD). The presence of FS groups on the tips is confirmed by x-ray photoelectron spectroscopy (XPS) and Fourier transform infrared spectroscopy (FTIR) tests. After lubrication of the tips through a fluorinated lubricant, the omniphobicity and repellent behaviour of the tips drastically enhanced which are revealed via static and hysteresis contact angle measurements. The repellency of the lubricant-infused pipette tips against physical adsorption is investigated through pipetting a food coloring dye as well as human blood samples and are compared to the untreated tips. The results show significantly less amount carryover residue when the lubricant-infused tips are utilized compared to commercially available ones. We also demonstrate the lubricant-infused tips reduce bacteria contamination of the inner lumen by 3 to 6-log (over 99%, depending on the tip size) after pipetting up and down the bacteria solution.

## Introduction

Fluid carryover in liquid handling devices can lead to experiment failure, measurement inaccuracies and sample loss. It is the major cause of cross-contamination in general scientific procedures such as bacteriological work, polymerase chain reaction (PCR), and radioimmunoassay^1–4^. For instance, in PCR amplification reactions in a criminal forensic lab, small amounts of DNA contamination could result in amplifying DNA to promote false positive identifications^5^. In such instances, an underlying cause of contamination could originate from the pipetting of substances with highly viscosities and low surface tensions which can stick to the plastic surface of the pipette, resulting in an improper ejection onto the next test sample^6–8^. Moreover, carryover contamination in pipetting can lead to erroneous volume determination, the need for changing tips, carbon footprint and costs associated with disposing of single use tips. In order to substantially minimize the effect of carryover, laboratory work areas need separate sets of supplies and equipment, such as pipettes, test-tube holders, and centrifuges^9–11^. Single-use tips with filters are usually recommended as the main strategy to prevent contamination originated form amplicons that accumulate within pipettor^10,12–14^. However, single-use tips are not applicable to automated robotic workstations with their use of fixed tips^15^. Techniques have currently been put in place for prevention of cross-contamination, through increasing well-to-well spacing and the prevention of involuntarily z-axes movements from robotic gripper during plate transfer^10,16^. In addition, present literature suggests fixed tips treated with robust washing routines can serve as a viable and effective alternative to disposable tips^16^. The issue of carryover contamination can be broadened towards other forms of laboratory equipment, such as the syringes and needles used in the removal of PCR products on the robotic arm of automated amplification systems^17^. A potential solution would involve surface modifications to laboratory equipment, namely pipette tips, such that the issue of sample carryover can be minimized.

Most pipette tips on the market are manufactured with hydrophobic polymers that repel high surface tension liquids like water. One of the most commonly used polymers to mold pipette tips is polypropylene due to its hydrophobicity, cost effectiveness, and availability. While hydrophobic and superhydrophobic surfaces are useful for repelling high surface tension liquids, they lack in performance when liquids with low surface tension or high viscosity are used. Hydrophobic adsorption of biomolecules to the tips can also arise inaccuracies and loss of sample during pipetting^18^.

To address this issue, the surface of the tip must be rendered omniphobic in order to repel different types of liquids and biofluids despite their cohesive force strengths^19–21^. A low dynamic angle hysteresis and a low sliding angle are generally characteristic of omniphobic surfaces^22–26^. Omniphobic surfaces can be fabricated using two general techniques: physical and chemical modifications^19,27–33^. Physical modification involves roughening the surface using methods such as nano particle deposition, lithographic imprinting and etching^34–36^. Chemical modification lies on the basis of decreasing the free energy of the surface of interest^1,19,20,37–41^. Several surface modification techniques that rely on altering the surface chemistry are known, some of the most prevalent include the use of fluorocarbon compounds or organosilanes as surface coatings^21,34,42–44^.

The growing demand for pipette tips with minimal sample surface adherence has led to the development of low retention pipette tips. Low retention pipette tips exhibit omniphobic surface properties thereby leading to minimal sample loss. One example involves using unique molding processes to incorporate fluoropolymer molecules into the surface of the pipette tips to achieve hydrophobic properties^45^. Texturing the pipette tips is another technique used by researchers to hydrophobically coat the pipette tips^46,47^. These methods, however, present negative aspects such as increased manufacturing complexity, high cost and lower effectiveness as compared to surface coating.

Here, we propose a simple and cost-effective process comprising fluorosilanization of pipette tips via chemical vapour deposition (CVD) method followed by lubrication to generate lubricant-infused tips with omniphobic properties. The fluorosilane (FS) layer and omniphobicity of the lubricant-infused tips have been characterized via x-ray photoelectron spectroscopy (XPS), Fourier transform infrared spectroscopy (FTIR), as well as static and hysteresis contact angle measurements. The efficacy of the modified tips in preventing carryover residue is demonstrated by pipetting different solutions such as food coloring dye, human recalcified blood, and bacteria solutions.

## Materials and methods

### Materials

Trichloro (1H, 1H, 2H, 2H-perfluorooctyl) silane (TPFS), perfluoroperhydrophenanthrene (PFPP), tryptic soy broth (TSB) (Sigma, Oakville, Canada), pipette tips composed of polypropylene (Diamed Lab Supplies Inc., Mississauga, Canada), red food coloring dye (A Preema Quality Product, Ingredients: Sodium Chloride and E122 Carmosine), and tween 20 (Sigma, Oakville, Canada) were used as received. Citrated human blood was generated from blood samples collected from healthy donors. All procedures were approved by the McMaster University Research Ethics Board. All methods were performed in accordance with the relevant guidelines and regulations. Informed consent was obtained from all the donors.

### Methods

**Figure 1:**
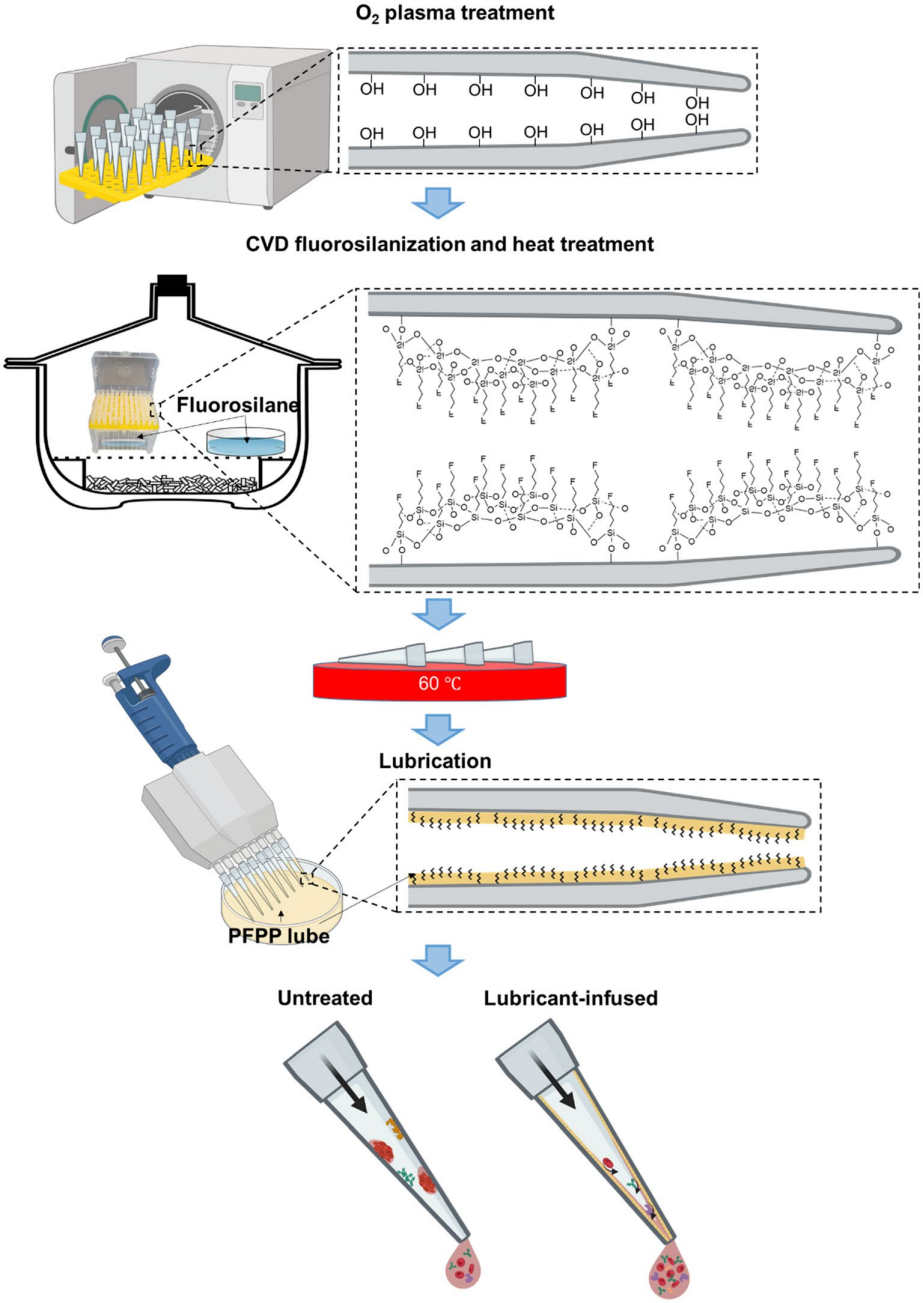
Schematic illustration of the treatment procedure along with the chemical structures of the treated tips at each step of the modification. Pipette tips were oxygen plasma treated followed by CVD treatment with TPFS and heat treatment at 60 °C overnight. The tips were subsequently lubricated via pipetting in and out PFPP lubricant. The lubricant layer can repel different types of biomolecules and solutions whereas in the commercially available tips (untreated tips), biomolecules and reagents can attach to the inner lumen of the tips causing inaccuracies in volumes and concentrations.

Pipette tips were put into a tip rack and placed in a plasma cleaner (PE-100, Plasma Etch). The tips were plasma treated at radio frequency (RF) of 150 kHz at 25 °C for 2 min using oxygen gas. Upon oxygen plasma-treatment, the pipette tips were placed in a desiccator connected to a vacuum pump. 200 µL of TPFS was pipetted onto a glass slide on a separate petri dish located across from the pipette tips rack. In addition, 100 µL of TPFS was also pipetted on glass slide located inside the rack (Fig. 1). The desiccator was vacuumed at a pressure of − 0.08 MPa, therefore initiating the CVD treatment. The silanization reaction took place over a period of 2.5 h at room temperature. After completion of CVD, pipette tips were heat treated at 60 °C overnight. In order to infuse lubricant in the fluorosilanized pipette tips, the PFPP lubricant was simply pipetted in and out after which, the tips were thoroughly washed with deionized (DI) water to remove the extra amount of the lubricant, leaving a thin layer of lubricant locked into the FS groups via Van der Waals forces.

FTIR (Bruker, Karlsruhe, Germany) was used to assess the surface chemical composition of the pipette tips before and after the treatment. During FTIR measurements, the air was considered the background, and the other spectra of the fluorosilanized and untreated tip surfaces were normalized based on this baseline. X-ray photoelectron spectroscopy (XPS) (PHI Quantera II, Biointerfaces Institute, McMaster University) was employed to further exhibit the FS layer formed on the pipette tips. Worth mentioning that the XPS test was performed two weeks after preparation of the tips to ensure the stability of the FS layer son the tips. High-resolution XPS was conducted to measure the atomic concentrations % of C, O, Na, Si, S, and F. Before FTIR and XPS analyses, pipette tips were cut to expose the inner surface for the tests, and thoroughly washed with ethanol.

Contact angles of the fluorosilanized and untreated were measured using 5 µL droplets of water, tween 20, and isopropanol. Water sessile drop contact angle measurements were performed at room temperature before and after each modification step using a Future Digital Scientific OCA20 goniometer (Garden City, NY), which was calibrated prior to each measurement. Hysteresis contact angle was carried out on the lubricant-infused tips and untreated tips using the needle method, where the needle was brought close to the tip and the advancing and receding contact angles were measured while the droplet was being injected and withdrawn from the surface.

It should be noted that all the characterization tests were performed at the inner lumen of 1 mL tips through cutting them in small pieces and exposing the inner surface. For each characterization test, 3 replicates were considered.

Stability of the lubricant inside the tips was investigated via measuring the weight of the tips in P200 tips after pipetting 200 μL lubricant up and down, washing to remove the excess amount of lubricant, as well as 3-time and 20-time pipetting up and down DI water while the pipette was set at 200 μL.

In the dye experiments, 10X serial dilutions of the red dye were performed in a well-plate to assess carryover of treated tips. The initial concentration of the dye was 1 mg mL^−1^. The dye was serially diluted in water containing 0.05% tween 20 using lubricant infused and untreated tips for comparison. For the purpose of the carryover evaluation, the pipette tips during the serial dilutions were not changed and the same pipette tip was implemented throughout all the dilution steps. The well-plate was analyzed using a plate reader (Tecan Infinite M1000) to find the absorbance values.

The interaction between blood components and lubricant-infused tips were assessed with recalcified blood. Citrated human blood was recalcified by CaCl_2_ diluted in HEPES at a concentration of 12.5 mM. For scanning electron microscopic (SEM) imaging (JSM‐7000 F), after pipetting up and down the recalcified blood, the tips were cut and fixed using 2% glutaraldehyde diluted in PBS. The tips were then incubated in 1% osmium tetroxide in 0.1 m sodium cacodylate buffer for an hour, followed by dehydration via a graded series of ethanol and critical point drying by Leica EM CPD300 dryer (Leica Mikrosysteme GmbH, Wien, Austria) using liquid CO_2_ flush. Before imaging, the samples were gold sputtered (Polaron Model E5100 sputter coater, Watford, Hertfordshire). High resolution SEM images of the tips after fluorosilanization were achieved via JEOL JSM-7000F. The samples were cut and mounted on a stub using carbon tape and silver paste, they were then coated using a sputter coater (Polaron model E1500, Polaron Equipment Ltd., Watford, Hertfordshire) with 10 nm of platinum for SEM.

Bacterial growth assay was done by streaking *Staphylococcus aureus* USA300 JE2 (MRSA)^48^ onto LB agar from frozen and allowed to grow overnight at 37 °C. Overnight cultures were then diluted in tryptic soy broth (TSB) supplemented with 0.4% glucose and 3% NaCl^49^. Concentrated MRSA bacterial suspensions with a concentration of 10^7^ CFU mL^−1^ were then prepared. Untreated tips were inoculated with MRSA by pipetting the bacterial suspension up and down, whereas treated tips were initially lubricated and then washed 20 times with DI water prior to inoculation with the bacterial suspension. Next, the tips were incubated with shaking for 20 min in tubes containing 5 mL of TSB at 37 °C. From this solution, 100 μL of each vortexed sample was taken in order to run a CFU assay by plating serial dilutions on TSB agar Petri dishes. The statistical analyses were performed by ANOVA test.

## Results and discussion

In order to uniformly coat the inner surface of the pipette tips composed of polypropylene with FS groups, the tips were first oxygen plasma treated for 2 min and then CVD fluorosilanized for 2.5 h using TPFS solution. During this process, hydrophilic terminals (trichlorosilane) of fluorosilane molecules bind to the plasma induced hydroxyls on the surface of the tips, resulting in self-assembled monolayers (SAMs) of FS with an umbrella-shaped structure in a way that the fluorine terminals are exposed on the surface (Fig. 1).

**Figure 2:**
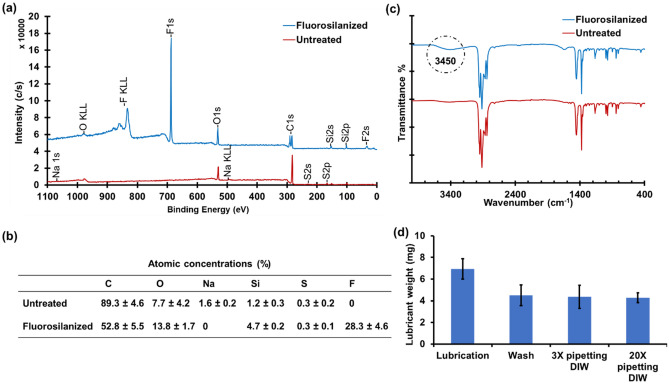
Characterization of the FS treated tips and lubricant stability**.** (**a**) XPS spectra of untreated tips and fluorosilanized tips. The emergence of F2*s*, F1*s*, and F KLL peaks are associated with the formation of FS layer inside the tips. (**b**) Atomic concentrations (%) of different elements at the inner surface of untreated and fluorosilanized tips resulted from high-resolution XPS analysis. (**c**) FTIR spectra of untreated pipette tips versus fluorosilanized tips. Circled broad peak in 3700–3200 cm^−1^ region indicates presence of Si–OH bonds**.** (**d**) Lubricant weight in the tips after lubrication, after washing off the excess amount of lubricant, and after pipetting DI water for 3 and 20 times. The amount of lubricant remained unchanged in the tips after multiple pipetting. The results are presented as means ± S.D.

To analyze the changes in the chemical composition of the FS treated pipette tips, XPS test was performed on the inner surface of fluorosilanized and untreated pipette tips (Fig. 2a). The emerged peaks at about 35 eV, 690 eV, and 830 eV in the fluorosilanized spectra are attributed to F2*s*, F1*s*, and F KLL bonds, respectively. These peaks indicate the formation of FS layer inside the tips using our developed CVD method. Figure 2b reveals the atomic concentrations of the elements present at the inner surface of untreated and fluorosilanized tips, using high-resolution XPS. After fluorosilanization, the concentration of F, Si, and O increased from 0 to 28.3%, 1.2 to 4.7%, and 7.7 to 13.8, respectively. This clearly illustrates the existence of fluorosilane tail groups as a result of FS SAM formation during the CVD process. Notable, the atomic concentrations of C and impurities such as Na was diminished due to the plasma etching of the tips and breaking apart the chemical bonds at the surface.

We also conducted FTIR test to further confirm the formation of FS layer inside the tips (Fig. 2c). The spectra of both fluorosilanized and untreated tips had a number of peaks in the absorption band between 800 and 1200 cm^−1^, specifically at 850 cm^−1^, 1000 cm^−1^, and 1200 cm^−1^ vibrations that are characteristic peaks for isotactic polypropylene. These absorption bands can be interpreted as vibrations of C–H, CH_2_ and CH_3_ groups in the polymer chain^50^. In the fluorosilanized tips, however, a broad absorbance band is present around the 3700–3200 cm^−1^. This could correspond to alcohols and phenols formed as a results of plasma treatment and hydrolysis of TPFS molecules^50^. Due to the relatively large penetration depth in FTIR, we could not precisely demonstrate the FS SAM related bonds via this method.

After fluorosilanization of the tips, they were lubricated by simply pipetting in and out a fluorinated lubricant (PFPP). The excess amount of the lubricant was then removed by extensively washing the tips via DI water. The remaining thin layer of the lubricant could be locked-in within the FS SAM through Van der Waals forces. This lubricant layer provides omniphobic properties and repellency at the inner lumen of the lubricant-infused tips. The lubricant, however, should remain in the tip after pipetting chemicals to preserve the omniphobicity. Considering several studies on instability of the lubricant layer^27,51–53^, we decided to evaluate the robustness of the lubricant-infused tips via weighing test. As can be seen in Fig. 2d, after lubrication of the tips, the weight of the lubricant was 6.9 mg. When the tips were washed and the excess lube was removed, the weight of the remaining lubricant inside the tip was reduced to 4.5 mg. This amount of lubricant was very stable inside the tip as after 20-time pipetting DI water, the weight of the lubricant in the tips remained unchanged. This also proves that the lubricant will not be introduced to the pipetted solution when the lubricant-infused tips are used. Taking into account the lubricated surface area of the tip and the weight and density of the PFPP lubricant (2.03 g/mL at 20 °C), we calculated that the thickness of the lubricant layer is approximately 7 μm.

Notably, according to the Gibbs free energy analysis, PFPP lubricant could naturally spread and form a lubricating layer even without the presence of FS SAM^27^. However, we realized that the lubricant at the surface of untreated tips is significantly less stable than the fluorosilanized tips. Figure 1S shows the weight of the remaining lubricant in the untreated tips after 20-time pipetting up and down DI water. The lower weight of lubricant in the untreated tips indicates that the lubricant was partially washed off the tip during the long-washing process. This could be due to the absence of fluorine layer and lack of van der Waals forces to lock-in the lubricant at the surface of the untreated tips. The remaining lubricant on the inner lumen of the untreated tips suggests that the untreated lubricated tips could potentially suppress carryover residue in the tips to an extent, eliminating the need for fluorosilanization. Nonetheless, without fluorosilanization, the lubricant might be stable for a short period of time and for long term applications, FS treatment is required.

**Figure 3:**
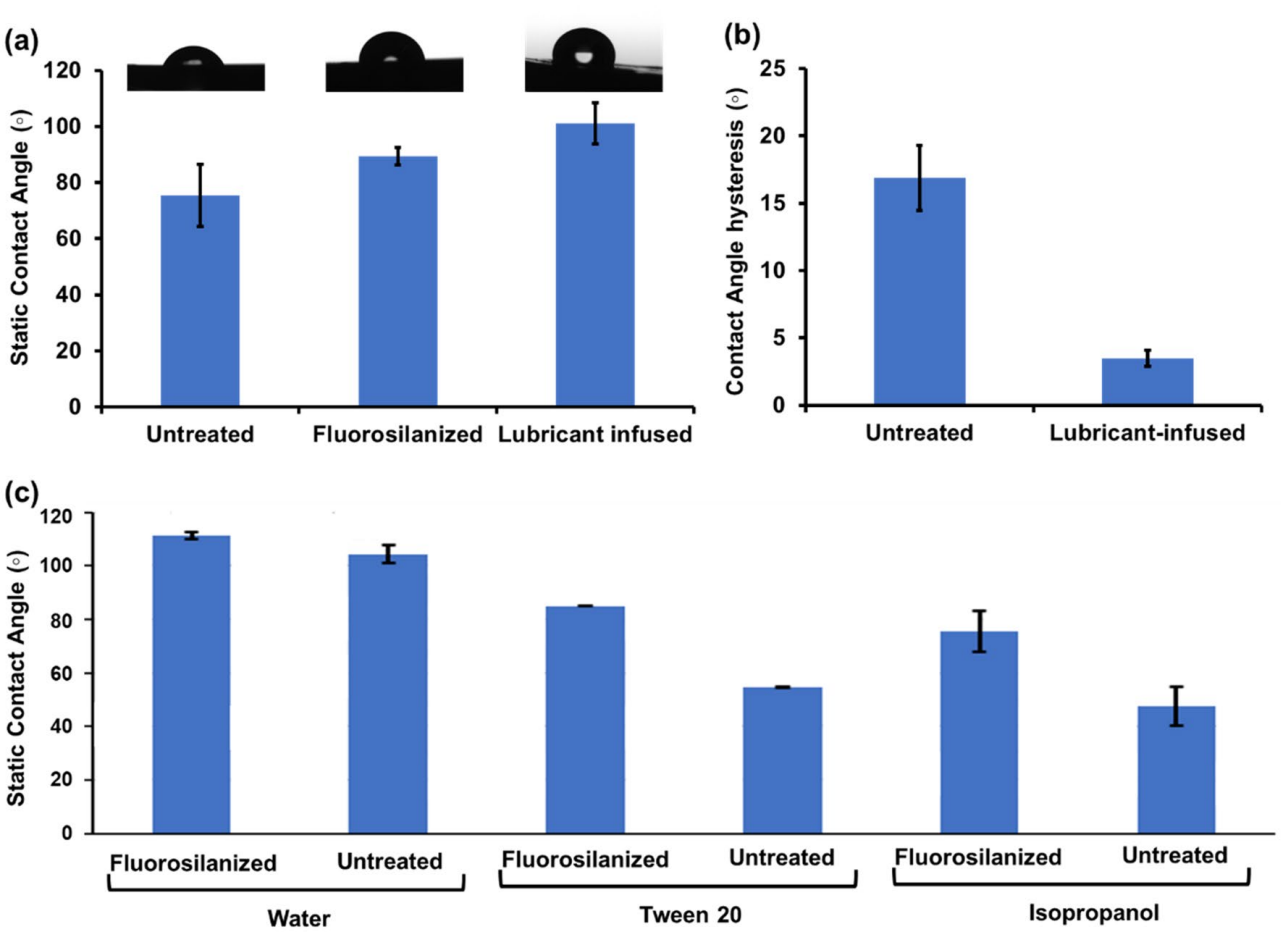
Contact angle measurements. (**a**) Static contact angle measurement of untreated, fluorosilanized, and lubricated pipette tip samples. (**b**) Contact angle hysteresis of untreated and lubricant-infused tips. (**c**) Static contact angle comparison between water, tween 20, and isopropanol measured using capillary pressure. All results are presented as means ± S.D.

To examine the relative hydrophobicity/hydrophilicity of the treated and untreated pipette tips, contact angle measurements were performed using a 5 µL droplet of deionized water. The static contact angle measurements of the untreated tips, fluorosilanized tips, and lubricant infused tips are shown in Fig. 3a. Untreated tips demonstrated a lower contact angle of 75 ± 11° implying low levels of hydrophobicity. It is worth noting that the standard deviation value of the untreated samples is relatively high due to the variation in the surface properties of the commercially available tips. Upon CVD treatment and before lubricant addition, the contact angle increased to 89 ± 3°. Finally, after addition of PFPP lubricant to the fluorosilanized tips, contact angles of water were demonstrated to increase to 101.7 ± 7°.

In the contact angle measurements, the tips were cut open and the measurement was done on the concave area to assess the hydrophobicity of the inner lumen. Due to the surface curvature of the pipette tip, measuring the contact angle of a liquid droplet on the tip using a tensiometer presents complications and might result in slight inaccuracy. As such, the contact angle of water, tween 20, and isopropanol as a low surface tension liquid were measured on treated and standard pipette tips using capillary rise method. Capillary rise can be utilized to compute the contact angle of the rising/dropping liquid using hydrostatics principles. Due to the close resemblance of the tips to a truncated cone shape, the capillary rise equation was modified to account for such capillary shape (Eq. 1):

1 $$\theta ={cos}^{-1}\left[\frac{\rho ghR}{2\gamma }\right]-\beta$$

where θ is the contact angle, ρ is the density of the liquid, g is the gravitational constant, h is the rise/drop height, R is the diameter of tube at the three-phase region, γ is the surface tension of the liquid, and β is the conical angle of tip.

As such, the contact angles of liquids with various surface tensions on the fluorosilanized and untreated tips were acquired to test the effect of surface modification on omniphobic properties of the tips (Fig. 3c). The contact angles of all three liquids were higher on the fluorosilanized tips compared to untreated tips. This is especially evident for the lower surface tension liquids such as the tween 20 and isopropanol. The increase in the contact angles of the treated samples highlight the higher surface repellency induced by the treatment. The contact angle of tween 20 was 30° higher on the fluorosilanized surface as compared to the standard while that of isopropanol was 28° higher. Since the tips exhibited hydrophobic properties even prior to surface treatment due to the properties of the material used to manufacture the tips, there was a smaller difference in contact angle of water on the treated surface with 6.9° increase using the capillary rise method and 40° increase using the optical tensiometer. Notable, the hydrophobic nature of the intact pipette tips was not fully appreciated in Fig. 3a which could be due to the fact that the surface properties of the tips somewhat changed when the tips were cut and flattened for the purpose of the contact angle measurement.

The drop retention force on the surface of the modified tips was assessed via hysteresis contact angle measurements (Fig. 3b). We exercised needle method to evaluate the dynamic contact angle due to the curvature of the tips. While the contact angle hysteresis of untreated tips was about 17 ± 2.5°, the contact angle hysteresis of lubricant-infused tips reached down to 3.5 ± 0.6°, showing great repellent behaviour of the developed tips.

As the developed lubricant-infused tips are intended to be versatile and simple, we assessed the possibility of incorporating air plasma treatment instead of oxygen plasma treatment in the fluorosilanization process. The tips were plasma treated under air for 5 min and subsequently FS CVD treated for 2.5 h followed by heat treatment at 60 °C overnight. The contact angles of fluorosilanized tips via air plasma treatment are compared with oxygen plasma treatment in Fig. 2S. The obtained contact angles from both plasma treatment protocols were identical, suggesting that air plasma treatment-which is substantially more accessible than oxygen plasma treatment in different laboratories, can perfectly be substituted in our fluorosilanization protocol.

Recent studies have been documented that textured fluorosilane layers are more effective in retaining lubricant^54^. We performed an SEM with high magnifications to investigate the effect of fluorosilanization on the surface topography of pipette tips (Fig. 3S). The results did not show any significant difference in the texture of untreated tips compared to fluorosilanized tips produced by either oxygen or air plasma treatment prior to fluorosilanization. Therefore, textured FS SAM coating could not be generated through our proposed strategy.

**Figure 4:**
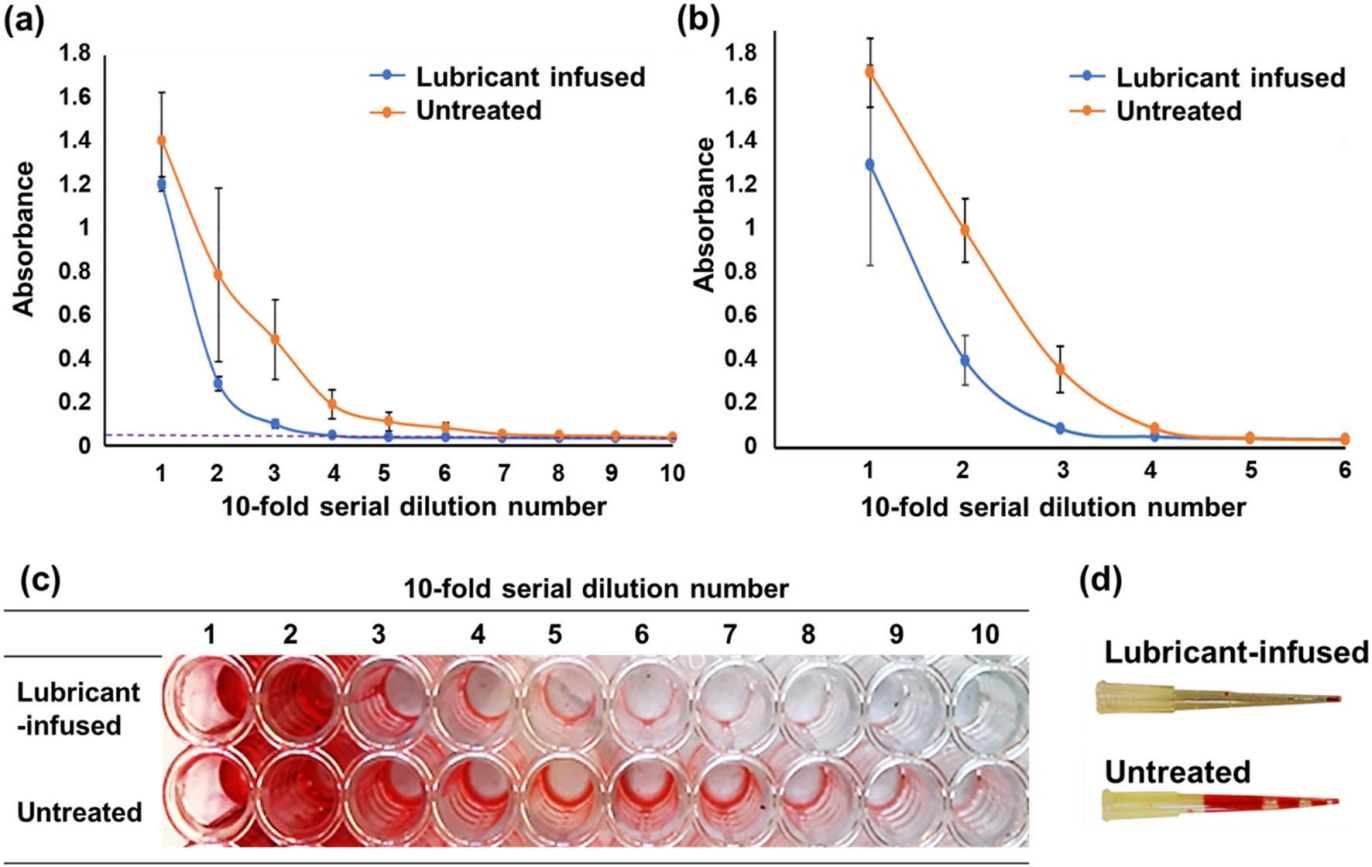
Absorbance of the red dye at different concentration after performing tenfold serial dilutions using (**a**) 10 μL and (**b**) 200 μL untreated tips compared to fluorosilanized lubricant infused tips. For each experiment a single tip was used for all the serial dilutions. Dashed line in (**a**) represents the absorbance at zero concentration of the red dye. The results are presented as means ± S.D. (**c**) Serial dilutions of the red dye in a well plate using single 10 μL untreated and lubricant infused tips. Untreated wells showed higher residue carryover of dye, as dilution did not occur effectively. (**d**) Comparison of the red dye attachment to 200 μL untreated tips and fluorosilanized lubricant infused tips.

In order to quantify the efficiency of lubricant-infused tips in preventing carryover residue and cross contamination during pipetting, tenfold serial dilutions of 1 mg mL^−1^ of a food coloring dye were performed in water containing 0.05% tween 20 using lubricant-infused and untreated tips. The same pipette tip was used to perform all the dilution steps to better illustrate the effect of dye residue inside the tip during the serial dilutions. Using absorbance measurements (at the wavelength of 512 nm), we quantified the results obtained from lubricant infused and untreated pipette tips in two sizes of 10 μL and 200 μL shown in Fig. 4a and b, respectively. The results of the dilutions indicate that the untreated tips have significantly higher carryover residue, and the dilutions did not occur efficiently. Hence, the more amount of dye that is seen at lower concentrations could be due to the dye residue that had remained onto the tip surface from the initial high concentrations and was carried to the low concentrations.

According to the Fig. 4a, b, the lubricant infused tips show a steeper decrease in absorbance per dilution number followed by a sooner levelling off to the diluent’s absorbance value (with zero concentration of the dye) that is indicated by dashed line in Fig. 4a. On the other hand, the untreated tips reveal higher absorbance levels even for higher dilution numbers which is a result of the sample adhering to the tip’s wall even after performing several dilutions. In addition, there is significantly higher variation observed with untreated tips compared to treated ones as indicated by large error bars shown in Fig. 4a, b. In Fig. 4c, the colour difference in the well-plate after performing the serial dilutions via the lubricant infused tips and untreated tips could be observed. We also performed a visual test at the tips after pipetting in and out the food coloring dye diluted in water containing 0.05% tween 20 at the concentration of 1 mg mL^−1^. As shown in Fig. 4d, the untreated 200 μL pipette tip showed a great amount of dye adsorption onto the tip, whereas the lubricant-infused tip maintained significantly lower amount of dye mostly at the bottom end of the tip after pipetting out the dye solution.

**Figure 5:**
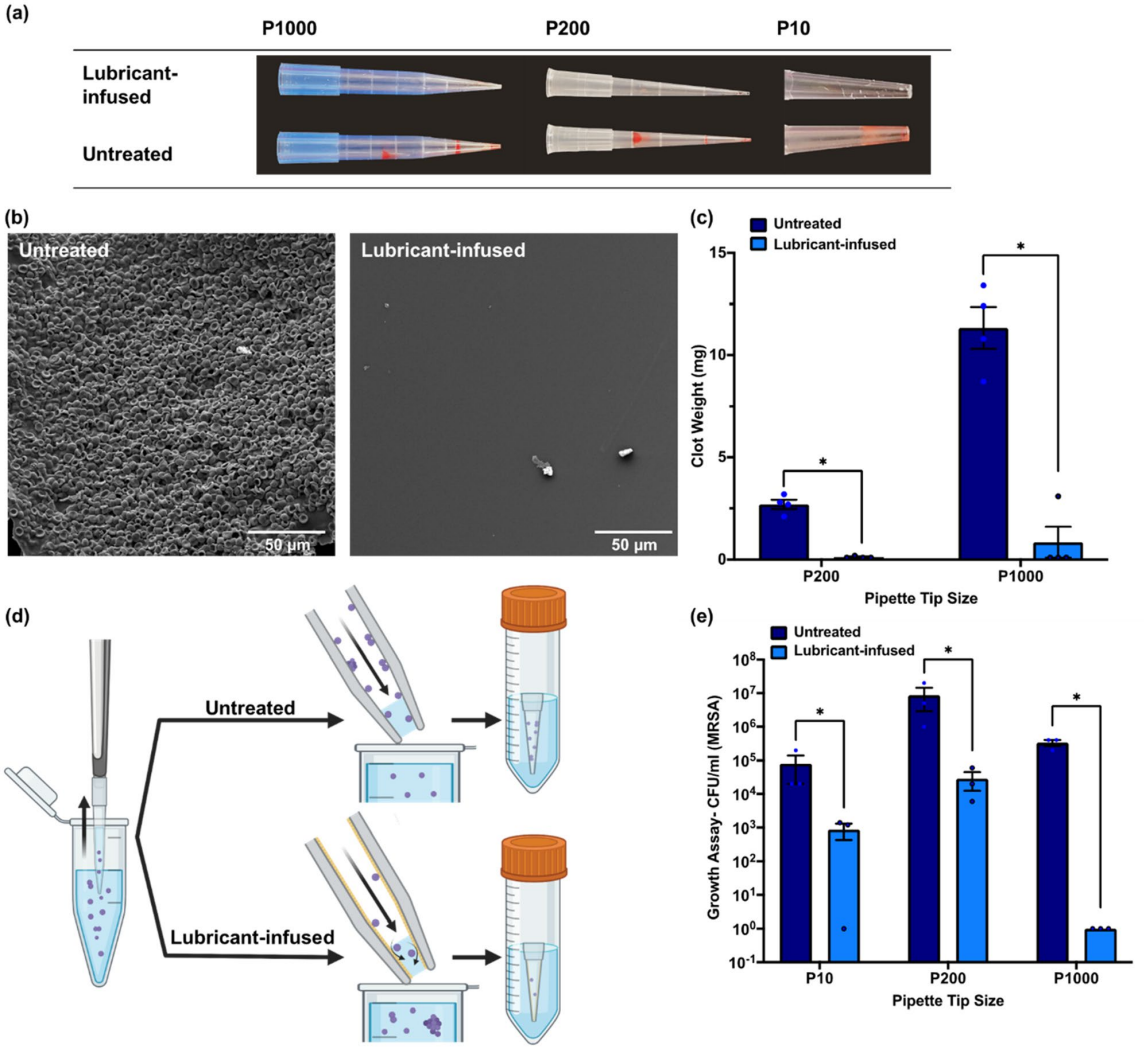
(**a**) Comparison of the clotting blood attachment to 10 μL (P10), 200 μL (P200), and 1000 μL (P1000) untreated tips and fluorosilanized lubricant infused tips after pipetting recalcified human blood. (**b**) SEM images of clot attached to the surface of the untreated tip compared to the fluorosilanized lubricant infused tip. (**c**) Clot weight in the untreated and lubricant-infused tips in two sizes of P200 and P1000. The amount of clot in the lubricant-infused tips was significantly lower than untreated tips (*P < 0.001). The results are presented as means ± S.D. (**d**) Schematic representation of bacteria assay. After pipetting up and down the MRSA bacteria suspension using both untreated and lubricant-infused tips, the tips were incubated in TSB media for 20 min. (**e**) Bacteria growth assay results achieved from the TSB media in which the tips were incubated. There is a significant difference in CFU/mL MRSA between the untreated and lubricant-infused tips for all the three sizes of P10, P200, and P1000 (*P < 0.001). The results are presented as means ± S.E.M.

In the next experiment, we used lubricant infused tips at different sizes of 10 μL (P10), 200 μL (P200), and 1 mL (P1000), to pipette in and out recalcified citrated human blood. As it is demonstrated in Fig. 5a, the lubricant infused tips in all sizes could effectively suppress blood adhesion and clot formation inside the tip in comparison with the untreated tips. SEM images in Fig. 5b also exhibits the presence of blood cells and clot on the surface of the untreated tips, while the lubricant infused modification significantly reduced the cell attachment and clot formation. We quantified the amount of clot formed inside the tips by measuring the weight of the clot. Figure 5c shows that the clot weight was significantly lower in P1000 and P200 lubricant-infused tips compared to the untreated tips. Due to the limitation in sensitivity of the weighing scale, we were not able to measure the clot weight in 10 uL tips. Overall, the results show that lubricant-infused tips are capable of repelling all blood cells, clotting factors, and other bio-species present in human whole blood.

Finally, we utilized our lubricant-infused tips to pipette MRSA bacteria solution at a concentration of 10^7^ CFU mL^−1^. After pipetting up and down the bacteria suspension using both lubricant-infused and untreated tips, the tips were submerged in 5 ml TSB and incubated in a shaking incubator for 20 min at 37 °C (Fig. 5d). Further, CFU assay was run using the TSB solutions to compare the amount of bacteria attachment to the lubricant-infused tips and untreated tips. The growth assay results (Fig. 5e) showed about 2-log, 3-log, and 6-log reduction in CFU/ml MRSA by using P10, P200, and P1000 lubricant-infused tips, respectively. The lubricant-infused tips could repel the majority of MRSA bacteria when the bacteria solution was pipetted out and only a few bacteria remained in the tip (Fig. 5d). The result of this study reveals that a large number of bacteria physically attach to the commercially available tips during pipetting bacteria suspensions, which can bring about variation in the bacteria concentration of the pipetted solution and cause experimental errors.

## Conclusions

Sample adherence to tip wall during pipetting activities introduces many problems downsizing experimental accuracy. Current techniques to reduce this effect are expensive and require modifications to the plastic materials used for manufacturing the pipette tips. This work proposes a simple surface modification technique to induce liquid/protein repellency on the inner and outer walls of the pipette tips. The proposed lubricant-infused tips with omniphobic properties are able to repel low and high surface tension liquids and reduce sample carryover residue in the tips. We observed significantly lower amount of dye, blood clot, and bacteria attachment to the inner surface of the lubricant-infused tips compared to the untreated tips. This technique can thus be used to minimize cross-contamination and increase efficiency by decreasing sample loss due to the carryover adherence.

## Supplementary Information

Supplementary Information.

## Data Availability

The datasets generated and analysed during the current study are available from the corresponding author on reasonable request.
