# Supplementary material for: Contamination and carryover free handling of complex fluids using lubricant-infused pipette tips
Source: Sci Rep. 2022 Aug 25;12:14486. doi: 10.1038/s41598-022-18756-x (PMC9411573; doi:10.1038/s41598-022-18756-x)
Supplement: Supplementary file 1 — Supplementary Information. [file 41598_2022_18756_MOESM1_ESM.docx]

**Contamination and carryover free handling of complex fluids using lubricant-infused pipette tips**

Amid Shakeri^a*^, Hanie Yousefi^ab^, Noor Abu Jarad^c^, Samer Kullab^a^, Dalya Al-Mfarej^a^, Martin Rottman^d^, Tohid Didar^*a^

^a^ Department of Mechanical Engineering, McMaster University, 1280 Main Street West, Hamilton, ON L8S 4L7, Canada

^b^ Leslie Dan Faculty of Pharmacy, University of Toronto, 144 College Street, Toronto, ON M5S 3M2, Canada

^c^ School of Biomedical Engineering, McMaster University, 1280 Main Street West, Hamilton, ON L8S 3L8, Canada

^d^ Department of Microbiology and Innovative Biomarkers Platform, Hôpital Raymond Poincaré (APHP), Laboratory of Infection & Inflammation U1173, School of Medicine Simone Veil, Versailles Saint-Quentin-en-Yvelines University, France

* To whom correspondence should be addressed. E-mails: [shakeria@mcmaster.ca](mailto:shakeria@mcmaster.ca) & [didar@mcmaster.ca](mailto:didar@mcmaster.ca),


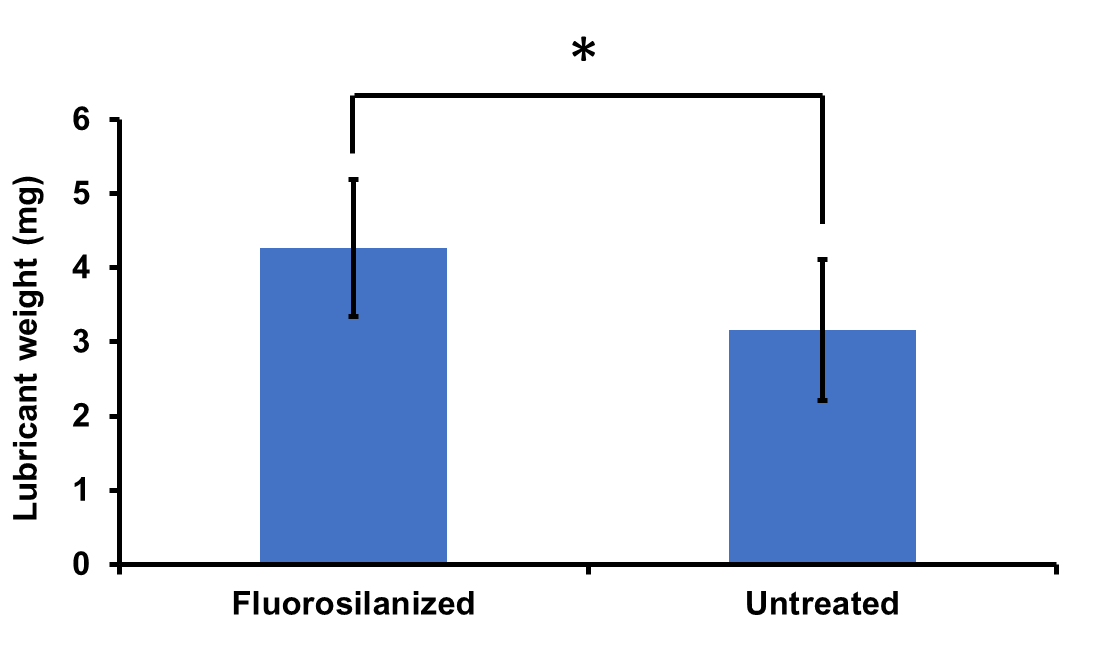


**Figure 1S.** Lubricant weight in the tips after lubrication followed by 20-time pipetting DI water. The remaining lubricant in the untreated tips was significantly less than the fluorosilanized tips (*P < 0.05), demonstrating that the lubricant was partially washed off the untreated tips during the 20-time pipetting process. The results are presented as means ± S.D.

**
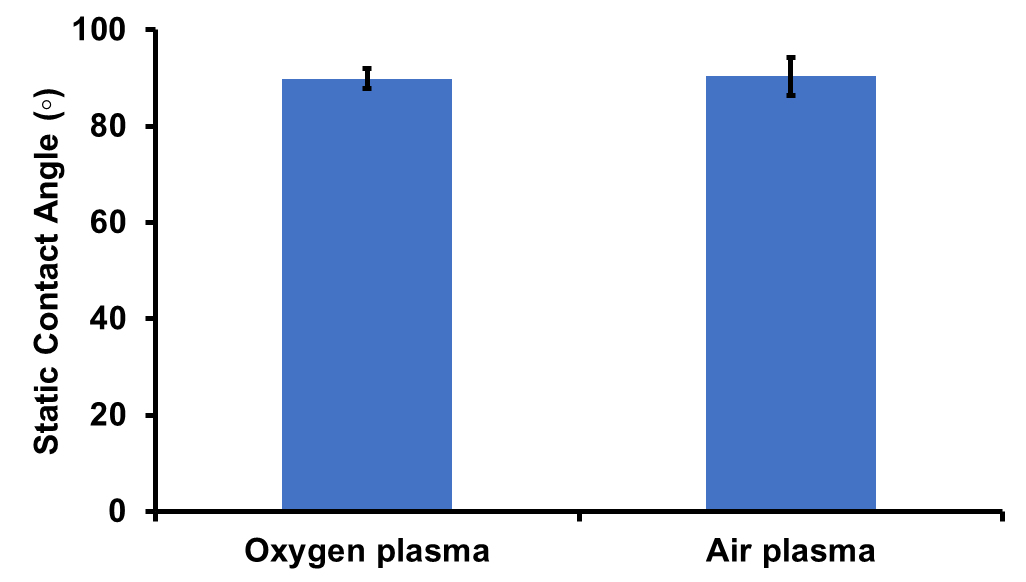
**

**Figure 2S.** Static contact angle of fluorosilanized tips produced by oxygen plasma treatment and air plasma treatment prior to FS CVD treatment. The results confirm the efficiency of air plasma treatment in the fluorosilanization process. The results are presented as means ± S.D.

**
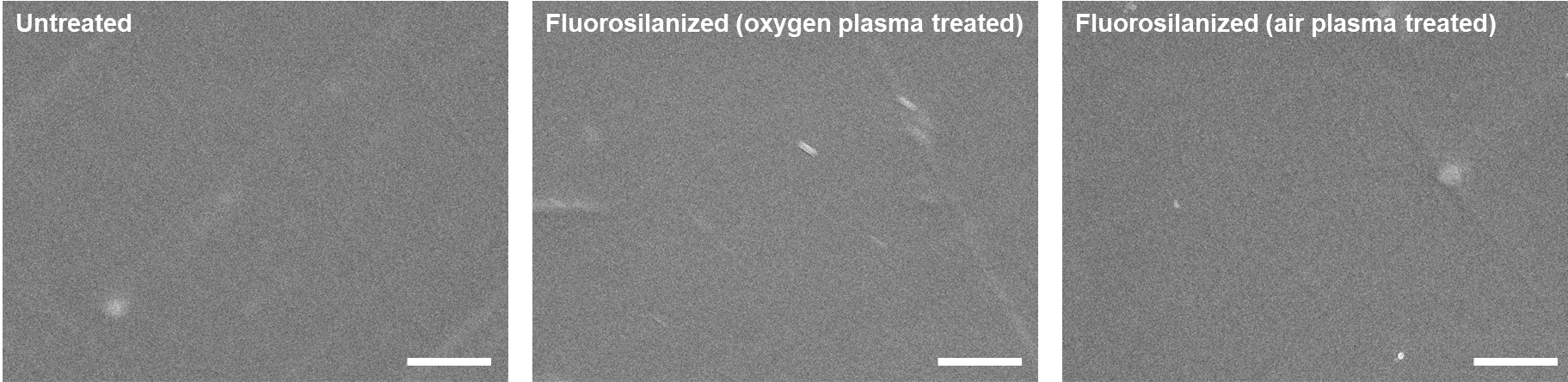
**

**Figure 3S.** SEM images of the inner surfaces of pipette tips. Fluorosilanization of pipette tips subsequent to oxygen or air plasma treatment did not significantly affect surface topography of the tips. Scale bar is 5 𝛍m.
